# Ethanol-induced changes in neurotrophic and immune genes are regulated by receptor-type protein tyrosine phosphatase β/ζ (RPTPβ/ζ) and microglial-neuronal interactions

**DOI:** 10.3389/fgene.2025.1634202

**Published:** 2025-08-22

**Authors:** María Aránzazu Penedo, Héctor Cañeque-Rufo, Esther Gramage, Gonzalo Herradón

**Affiliations:** ^1^ Department of Health and Pharmaceutical Sciences, School of Pharmacy, Universidad San Pablo-CEU, CEU Universities, Madrid, Spain; ^2^ Red de Investigación en Atención Primaria de Adicciones, Instituto de Salud Carlos III, MICINN and FEDER, Madrid, Spain; ^3^ Department of Chemistry and Biochemistry, School of Pharmacy, Universidad San Pablo-CEU, CEU Universities, Madrid, Spain; ^4^ Instituto de Estudios de las Adicciones, Universidad San Pablo-CEU, CEU Universities, Madrid, Spain

**Keywords:** alcohol, RPTPβ/ζ, co-culture, neuroinflammation, pleiotrophin, midkine

## Abstract

Microglial cells are key mediators of ethanol-induced neuroinflammation through the release of proinflammatory cytokines and activation of Toll-like receptors. Recently, the signaling pathway initiated by the interaction of the neurotrophic factors pleiotrophin (PTN) and midkine (MK) with receptor-type protein tyrosine phosphatase β/ζ (RPTPβ/ζ) has emerged as a pharmacological target in ethanol-induced neuroinflammatory and neurodegenerative processes. However, the underlying molecular mechanisms remain unclear. In this study, we developed a human co-culture system composed of differentiated SH-SY5Y neuronal cells and HMC3 microglial cells to simulate microglial-neuronal interactions during ethanol exposure. In HMC3 cells, *PTN* mRNA expression levels were significantly upregulated by ethanol exposure, whereas *MK* levels were not altered. In contrast, ethanol exposure caused a significant downregulation of *MK* expression in co-cultures. In general, ethanol increased the expression of inflammatory genes in monocultures of HMC3 cells but not in SH-SY5Y cells. In addition, ethanol exposure caused a highly significant upregulation of *TLR3* and *TLR4* in HMC3 cells, which was absent in co-cultures. We also observed a significant attenuation of ethanol-induced increases of inflammatory markers such as *IL-1β* and *CCL2 in* co-cultures, indicating the regulatory role of neuronal-microglial interactions. In conclusion, our study provides novel insights into the modulatory actions of microglial-neuronal interactions in ethanol-induced neuroimmune responses and suggests the therapeutic potential of the PTN/RPTPβ/ζ signaling pathway to prevent the deleterious effects of alcohol on the brain.

## 1 Introduction

Alcohol consumption remains a critical public health concern due to its profound and lasting effects on the brain. Excessive intake has been shown to exert direct neurotoxic effects and to trigger persistent neuroinflammatory processes that contribute to neurodegeneration (Pascual et al., 2021). Neuroimmune responses, particularly those mediated by sustained microglial activation and the synthesis and release of pro-inflammatory cytokines, play a central role in this chronic inflammatory state, which can persist even after alcohol withdrawal (Crews et al., 2024; Crews et al., 2011). Over time, this environment may disrupt synaptic plasticity and neurogenesis, leading to cognitive and motor impairments, and contributing to the onset and progression of various neurodegenerative disorders, including alcohol-related cognitive decline (Crews, 2012; Pascual et al., 2018; Walter and Crews, 2017).

Understanding the molecular mechanisms involved in alcohol-induced neuroinflammation in humans presents considerable ethical and methodological challenges. Numerous studies have reported alterations at both the cellular and microenvironmental levels of the central nervous system (CNS), as well as behavioral changes associated with alcohol use disorder (AUD) (Pascual et al., 2018; Hartung, 2009; Walter and Crews, 2017). However, most of these investigations have focused on descriptive characterizations, and the specific mechanisms driving these changes are still poorly understood. In this context, *in vitro* cellular models offer a valuable alternative for exploring molecular pathways and evaluating novel therapeutic strategies (Goshi et al., 2020). Particularly promising are co-culture systems, which recreate physiologically relevant interactions between key CNS cell types (Gresa-Arribas et al., 2012; Roqué and Costa, 2017). Among them, the interplay between dopaminergic neurons and microglia appears to be especially important in mediating the inflammatory and neurotoxic responses to ethanol exposure (Alfonso-Loeches et al., 2010; Correa et al., 2013; Kraft and Harry, 2011).

Recently, the signaling pathway initiated by the interaction of pleiotrophin (PTN) and midkine (MK) with receptor-type protein tyrosine phosphatase β/ζ (RPTPβ/ζ), also known as PTPRZ1, has emerged as a molecular axis of interest in neuroinflammatory and neurodegenerative processes. PTN and MK are neurotrophic factors and endogenous inhibitors of the tyrosine phosphatase activity of RPTPβ/ζ (Herradon et al., 2019). This receptor regulates interactions between glial and neuronal cells (Del Campo et al., 2021), and its pharmacological modulation is being explored as a promising strategy to mitigate alcohol-induced neuropathology (Fernández-Calle et al., 2018; Galán-LLario et al., 2023a; Galán-LLario et al., 2023b; Galán-LLario et al., 2024; Rodríguez-Zapata et al., 2023). To further characterize the functional roles of RPTPβ/ζ, our group designed and synthesized MY10, a selective inhibitor that interacts with its intracellular PD1 domain, effectively inactivating the tyrosine phosphatase activity of RPTPβ/ζ (Pastor et al., 2018).

Preclinical studies have shown that treatment with MY10 reduces alcohol consumption and blocks conditioned place preference, suggesting a targeted action on alcohol-related reward mechanisms (Calleja-Conde et al., 2020; Fernández-Calle et al., 2018). Additionally, MY10 modulates gene expression in the prefrontal cortex, likely through the regulation of PTN and MK signaling pathways (Fernández-Calle et al., 2017; Fernández-Calle et al., 2020; Herradón and Pérez-García, 2014). In addition, MY10 has demonstrated significant neuroprotective effects against ethanol-induced neuroinflammation. Recent findings demonstrate that MY10 also prevents ethanol-induced impairments in adolescent hippocampal neurogenesis, attenuates microglial reactivity, and modulates the organization of perineuronal nets (PNNs), with some effects showing sex-dependent variability (Galán-Llario et al., 2023a; Galán-LLario et al., 2023b; Galán-LLario et al., 2024). Further supporting its mechanism of action, PTN overexpression has been found to reproduce both the neuroprotective and ethanol-suppressive effects of MY10 (Galán-Llario et al., 2024).

The present study uses a human *in vitro* co-culture model composed of dopaminergic neurons and microglia, designed to simulate ethanol-induced neuroinflammation and to characterize the role of RPTPβ/ζ in this process. This model enables the study of neuron–glia crosstalk, a fundamental component of ethanol-driven neuroinflammatory processes, and allows the evaluation of pharmacological candidates such as MY10. The findings provide substantial evidence that neuron–microglia interactions critically influence the responses to ethanol, representing a meaningful advancement in the development of physiologically relevant platforms for investigating alcohol-induced neuropathology.

## 2 Materials and methods

### 2.1 Cell cultures

Human SH-SY5Y neuroblastoma cells and HMC3 human microglial cells (generously provided by Dr. Marta del Campo, ATCC) were used in this study.

#### 2.1.1 SH-SY5Y cells

**FIGURE 1:**
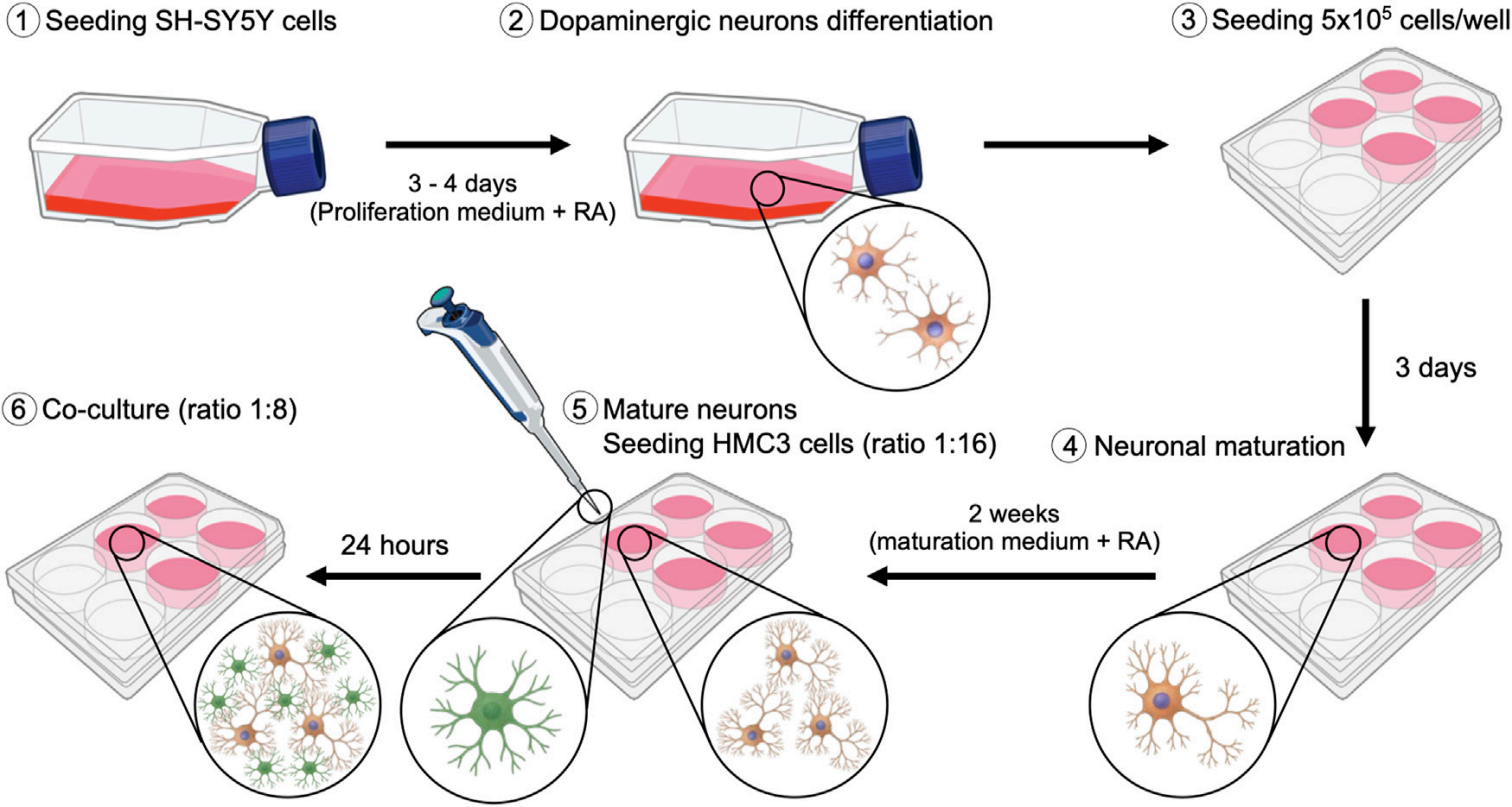
Schematic representation of the protocol implemented for the establishment of the SH-SY5Y and HMC3 co-culture.

SH-SY5Y cells were cultured in T-75 flasks at 37°C in a humidified atmosphere containing 5% CO_2_. To induce dopaminergic neuronal differentiation (Figure 1), cells were treated with 1:500 µM retinoic acid (RA, Thermo Fisher, United States) for 3–4 days in proliferation medium consisting of Dulbecco’s Modified Eagle’s Medium (DMEM, Biowest, France) supplemented with 10% Fetal Bovine Serum (FBS, Sigma-Aldrich, United States) and 1% penicillin/streptomycin (100 U/ml-100 μg/mL; Gibco, United States). After this initial phase, 5 × 10^5^ SH-SY5Y cells/well were seeded into 6-well treated culture plates (WVR, United States) and maintained additionally for 7 days in DMEM proliferation medium with RA. Medium was replaced every 2–3 days to maintain optimal differentiation conditions. Neuronal maturation was subsequently achieved by incubating cells for 14 days in maturation medium composed of Neurobasal (Gibco, United States), B-27 supplement (Gibco, United States), and 1% penicillin/streptomycin (Sigma-Aldrich, United States) with RA.

#### 2.1.2 HMC3 cells

5 × 10^5^ HMC3 microglial cells/well were cultured in 6-well plates using proliferation medium and maintained at 37°C in a humidified incubator with 5% CO_2_.

#### 2.1.3 SH-SY5Y and HMC3 co-culture

Once SH-SY5Y cells had ceased proliferation and exhibited mature neuronal morphology, HMC3 microglial cells were added to the culture at an initial ratio of 1:16 (HMC3:SH-SY5Y). The co-culture was maintained in neuronal maturation medium for 24 h to allow HMC3 cells to adhere and establish cellular contacts and to acclimate to promote adaptation. Subsequently, HMC3 cells were permitted to proliferate within the co-culture until a final ratio of 1:8 (HMC3:SH-SY5Y) was reached.

### 2.2 Immunofluorescence

Immunofluorescence staining assays were performed to confirm the SH-SY5Y and HMC3 co-culture (see Supplementary Figure S1). Cells were washed with phosphate-buffered saline (PBS, pH 7.4) and *fixed with 4% paraformaldehyde for 5 min at room temperature*. Cells were then permeabilized and blocked using a solution containing 10% Normal Goat Serum (NGS, Abcam, United States) and 10% Bovine Serum Albumin (BSA, Sigma-Aldrich, United States), and 0,2% Triton X-100 in PBS for 1 h. Neuronal cells were identified using a primary antibody against MAP-2 (Ab221693, Abcam, United Kingdom), while microglial cells were labeled with an anti-IBA1 antibody (Ab5076, Abcam, United Kingdom). Secondary antibody incubation was carried out using Alexa Fluor 488-conjugated anti-rabbit IgG (A21206, Thermo Fisher, United States) and Alexa Fluor 555-conjugated anti-goat IgG (705-565-147, Jackson Immuno Research, United Kingdom). Nuclear staining was performed with DAPI (D1306, Invitrogen, United States). Coverslips were mounted with mounting medium and imaged using a fluorescence microscope (Leica DM5500).

### 2.3 Determination of the expression levels of neurotrophic and immune genes

Cultures were exposed to 100 mM ethanol for 24 h, using a 4% ethanol chamber to maintain ethanol concentration (Coleman, et al., 2017). Three experimental groups were evaluated: SH-SY5Y monoculture, HMC3 monoculture, and SH-SY5Y + HMC3 co-culture. To evaluate the potential effects of RPTPβ/ζ inhibition, cultures were treated with MY10 at concentrations of 0.1 µM, 1 μM, and 10 μM, diluted in DMSO (final concentrations range: 0.0005%–0.05%) (Sigma-Aldrich, United States). Control groups received as vehicle 0.05% DMSO. Each experimental condition was tested in five independent biological replicates, with three technical replicates per group.

Following treatment, adherent cells were washed with PBS (pH 7.4) and lysed using TRIzol Reagent (Thermo Fisher, United States). Total RNA was isolated using the RNeasy Mini Kit (Qiagen, Germany), following the manufacturers protocol. First-strand cDNA was synthesized using the First-Strand cDNA Synthesis Kit (NZYTech, Portugal). Quantitative real-time PCR (qPCR) analysis was performed using the SYBR Green detection method (#1725272, Bio-Rad, United States) on a CFX96 Real-Time PCR Detection System (Bio-Rad, United States). The relative expression of each target gene was normalized using three housekeeping genes: RPL30, HPRT, and PKM. Primer sequences designed and used for amplification are listed in Table 1.

**TABLE 1:** Primer sequences used for qPCR analysis.

Gen	Forward Primer 5’-3’	Reverse Primer 5’-3’
*RPL30*	AAG​ACG​AAA​AAG​TCG​CTG​GA	AAA​GCT​GGG​CAG​TTG​TTA​GC
*HPRT*	CAG​GCC​AGA​CTT​TGT​TGG​AT	TTG​CGC​TCA​TCT​TAG​GCT​TT
*PKM*	GGTTCGGAGGTTTGATGA	GGC​TTC​TTG​ATC​ATG​CTC​T
*PTN*	ACA​ATG​CCG​AAT​GCC​AGA​AG	AGG​TTT​GGG​CTT​GGT​CAG​TT
*MDK*	TTC​CTC​CTC​CTC​ACC​CTC​C	TCC​TTC​TTC​CAG​TTG​CAG​GG
*HMGB1*	TAT​GGC​AAA​AGC​GGA​CAA​GG	TTT​GGG​CGA​TAC​TCA​GAG​CA
*CCL2*	CAT​GAA​AGT​CTC​TGC​CGC​C	GGT​GAT​TCT​TCT​ATA​GCT​CGC​G
*iNOS*	GCT​GTG​CTC​CAT​AGT​TTC​CAG	GGT​GAT​GCT​CCC​AGA​CAT​G
*TNFα*	GAA​CCC​CGA​GTG​ACA​AGC​C	AGG​ACC​TGG​GAG​TAG​ATG​AGG
IL-1β	ACA​CAT​GGG​ATA​ACG​AGG​CT	ACG​CAG​GAC​AGG​TAC​AGA​TT
*TLR3*	GAA​CCT​CCA​GCA​CAA​TGA​GC	TGA​CAA​GCC​ATT​ATG​AGA​CAG​A
*TLR4*	CAA​AAT​CCC​CGA​CAA​CCT​CC	AGT​CCA​GAA​AAG​GCT​CCC​AG
*TLR7*	CAC​TCC​ATG​CCA​TCA​AGA​AAG​T	TGG​AAT​GTA​GAG​GTC​TGG​TTG​A

*RPL30*: Ribosomal protein L30; *HPRT*: Hypoxanthine-Guanine Phosphoribosyltransferase; *PKM*: pyruvate kinase M1/2; *PTN*: Pleiotrophin; *MK*: Midkine; *HMGB1*: high mobility group box 1; *CCL2*: C-C motif Chemokine ligand 2; *iNOS*: Inducible Nitric Oxide Synthase; *TNFα*: tumor necrosis factor α; *IL-1β*: Interleukin 1 beta; TLR3: toll-like receptor 3; *TLR4*: toll-like receptor 4; *TLR7*: toll-like receptor 7.

### 2.4 Statistical analysis

All statistical analyses were performed using GraphPad Prism version 10 (GraphPad Software, United States). Data are presented as mean ± standard error of the mean (SEM). T-Student tests were used for targeted analysis of genes associated with the RPTPβ/ζ pathway. For experiments involving multiple conditions, two-way ANOVA were conducted considering ethanol exposure and MY10 treatment as variables, followed by Bonferroni’s *Post-hoc* correction for multiple comparisons. A *P*-value <0.05 was considered statistically significant.

## 3 Results

### 3.1 Modulation of the components of the PTN/MK/RPTPβζ axis following ethanol exposure

**FIGURE 2:**
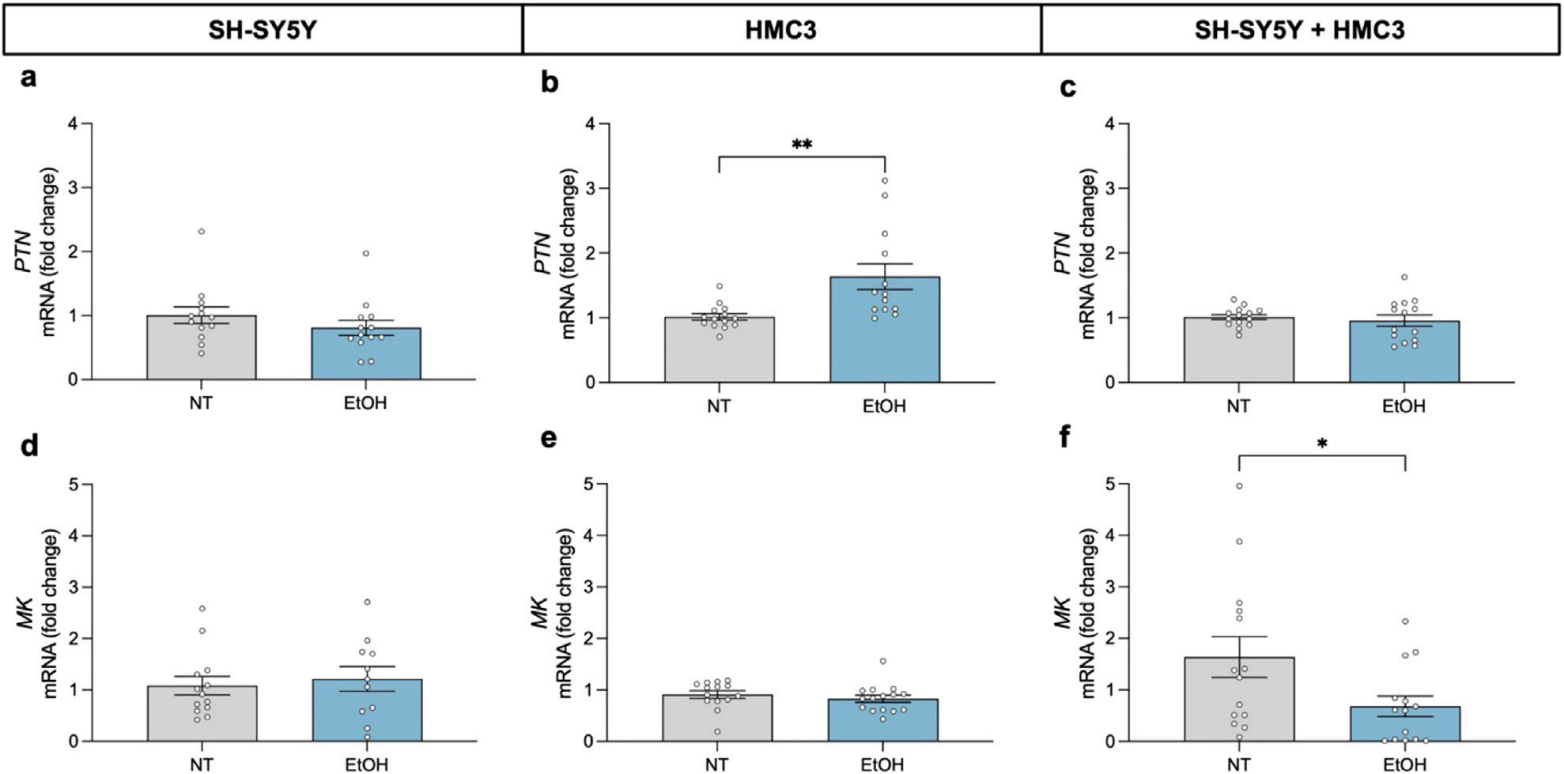
Effect of 24-h ethanol exposure (100 mM) on *PTN and MK* expression in neurons, microglia and co-cultures. *PTN* mRNA expression in SH-SY5Y cells **(a)**, HMC3 cells **(b)**, and co-culture **(c)**. *MK* mRNA expression in SH-SY5Y cells **(d)**, HMC3 cells **(e)**, and co-culture **(f)**. Data are presented as mean ± SEM. *P < 0,05; **P < 0,01. NT: no treatment; EtOH: ethanol.

We evaluated the transcriptional response of the RPTPβζ endogenous ligands *PTN* and *MK*, after 24-h exposure to 100 mM ethanol in three *in vitro* cellular models (Figure 2). Our results revealed that ethanol did not induce significant changes in the expression levels of these cytokines in SH-SY5Y dopaminergic neurons (Figures 2a,d). However, in HMC3 cells, *PTN* mRNA expression levels were significantly upregulated compared to untreated controls (Figure 2b; *t* = 3.149; *P* = 0.0042). In contrast, *MK* mRNA expression levels were not altered by ethanol exposure in microglial cells (Figure 2e). Interestingly, in the neuron-microglia co-culture system, ethanol exposure caused a significant downregulation of *MK* expression (Figure 2f; *t* = 2.162; *P* = 0.04), without altering *PTN* levels (Figure 2c). The data suggest that neuron-microglia interactions modulate the microglial response to ethanol and highlight the importance of cellular crosstalk in shaping proinflammatory signaling.

### 3.2 Impact of pharmacological inhibition of RPTPβζ by MY10 on ethanol-induced neuroinflammation

**FIGURE 3:**
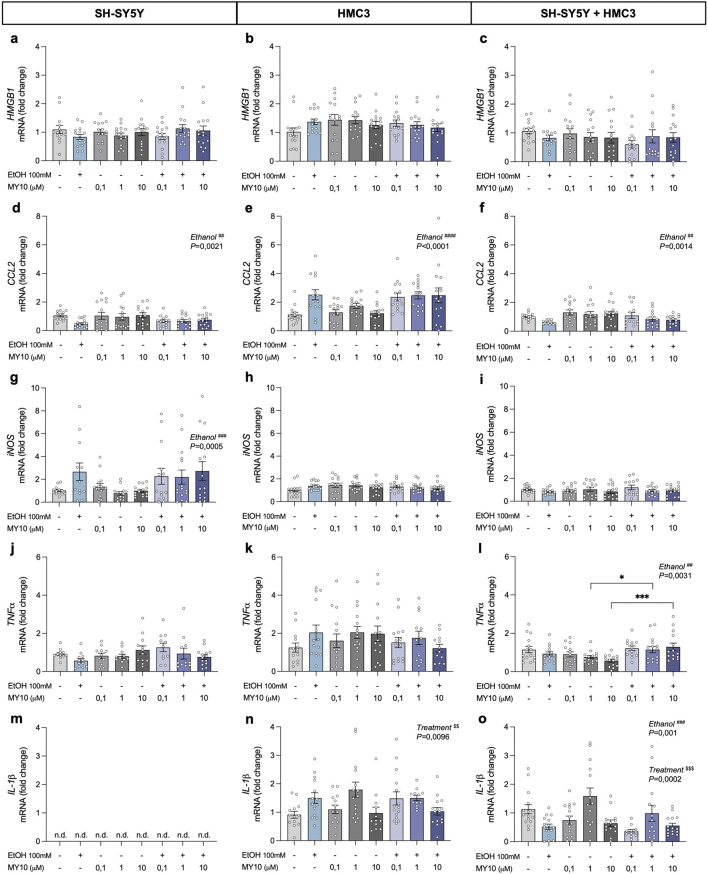
Effect of 24-h ethanol exposure (100 mM) and MY10 treatment on the expression of inflammatory genes in neurons, microglia and co-cultures. *HMGB1* mRNA expression in SH-SY5Y cells **(a)**, HMC3 cells **(b)**, and co-culture **(c)**. *CCL2* mRNA expression in SH-SY5Y cells **(d)**, HMC3 cells **(e)**, and co-culture **(f)**. *iNOS* mRNA expression in SH-SY5Y cells **(g)**, HMC3 cells **(h)**, and co-culture **(i)**. *TNFα* mRNA expression in SH-SY5Y cells **(j)**, HMC3 cells **(k)**, and co-culture **(l)**. *IL-1β* mRNA expression in SH-SY5Y cells **(m)**, HMC3 cells **(n)**, and co-culture **(o)**. Data are presented as mean ± SEM. *P < 0.05, ***P < 0.001. ^##^ *P* < 0.01, ^###^ *P* ≤ 0.001, ^####^ *P* < 0.0001 for a significant effect of EtOH exposure. ^$$^ *P* < 0.01, ^$$$^ *P* < 0.001 for significant effect of MY10 treatment. EtOH: ethanol.

To investigate the potential modulatory actions of MY10 on ethanol effects, we analyzed the expression of key proinflammatory genes in SH-SY5Y cells, HMC3 cells, and the co-culture of both in response to ethanol and different concentrations of MY10 (0.1 μM, 1 μM, and 10 μM) (Figure 3). Two-way ANOVA did not reveal significant effects of ethanol or the treatment with MY10 on the mRNA expression levels of *HMGB1* in any of the cell systems evaluated (Figures 3a–c). Interestingly, ethanol exposure significantly affected *CCL2* mRNA levels (Figures 3d–f). Specifically, ethanol caused a significant upregulation of *CCL2* levels in HMC3 microglial cells (*F* (1,107) = 29.62; *P* < 0.0001; Figure 3e). However, a significant downregulation was observed in SH-SY5Y neuronal cells (*F* (1,110) = 9.931; *P* = 0.0021; Figure 3d), and in the co-culture of neurons and microglia (*F* (1,106) = 10.72; *P* = 0.0014; Figure 3f). The two-way ANOVA performed with *iNOS* mRNA levels data only rendered a significant effect of ethanol exposure in SH-SY5Y cells (*F* (1,104) = 12.9; *P* = 0.0005), in which *iNOS* expression was upregulated by ethanol exposure (Figure 3g).

Additionally, we did not observe significant effects of ethanol exposure or the treatment with MY10 on *TNFα* mRNA levels in monocultures (Figures 3j,k). However, in the co-culture system (Figure 3l), two-way ANOVA showed a significant effect of ethanol exposure on the mRNA expression levels of *TNFα* (*F* (1,105) = 9.192; *P* = 0.0031), together with a significant interaction between ethanol and MY10 treatment (*F* (3,105) = 3.902; *P* = 0.0109). Treatment with MY10 tended to reduce the mRNA expression levels of *TNFα*, which was not observed in the groups treated concomitantly with MY10 and ethanol (Figure 3l).

Importantly, *IL-1β,* which was only expressed in microglia cells, showed contrasting profiles (Figures 3m–o). *IL-1β* mRNA expression in HMC3 cells (Figure 3n) was significantly upregulated by treatment with MY10 (*F* (3,101) = 4.015; *P* = 0.0096) and the same tendency was observed after ethanol exposure. In contrast, the two-way ANOVA performed with the data from the co-cultures of neurons and microglia revealed significant effects of the treatment with MY10 (*F* (3,106) = 7.307; *P* = 0.0002) and of ethanol exposure (*F* (1,106) = 11.42; *P* = 0.001). In the co-cultures, ethanol exposure decreased *IL-1β mRNA expression levels, which seemed to be only reverted by* 1 μM MY10 (Figure 3o).

### 3.3 Effect of MY10 on toll-like receptors expression (TLRs) after ethanol exposure

**FIGURE 4:**
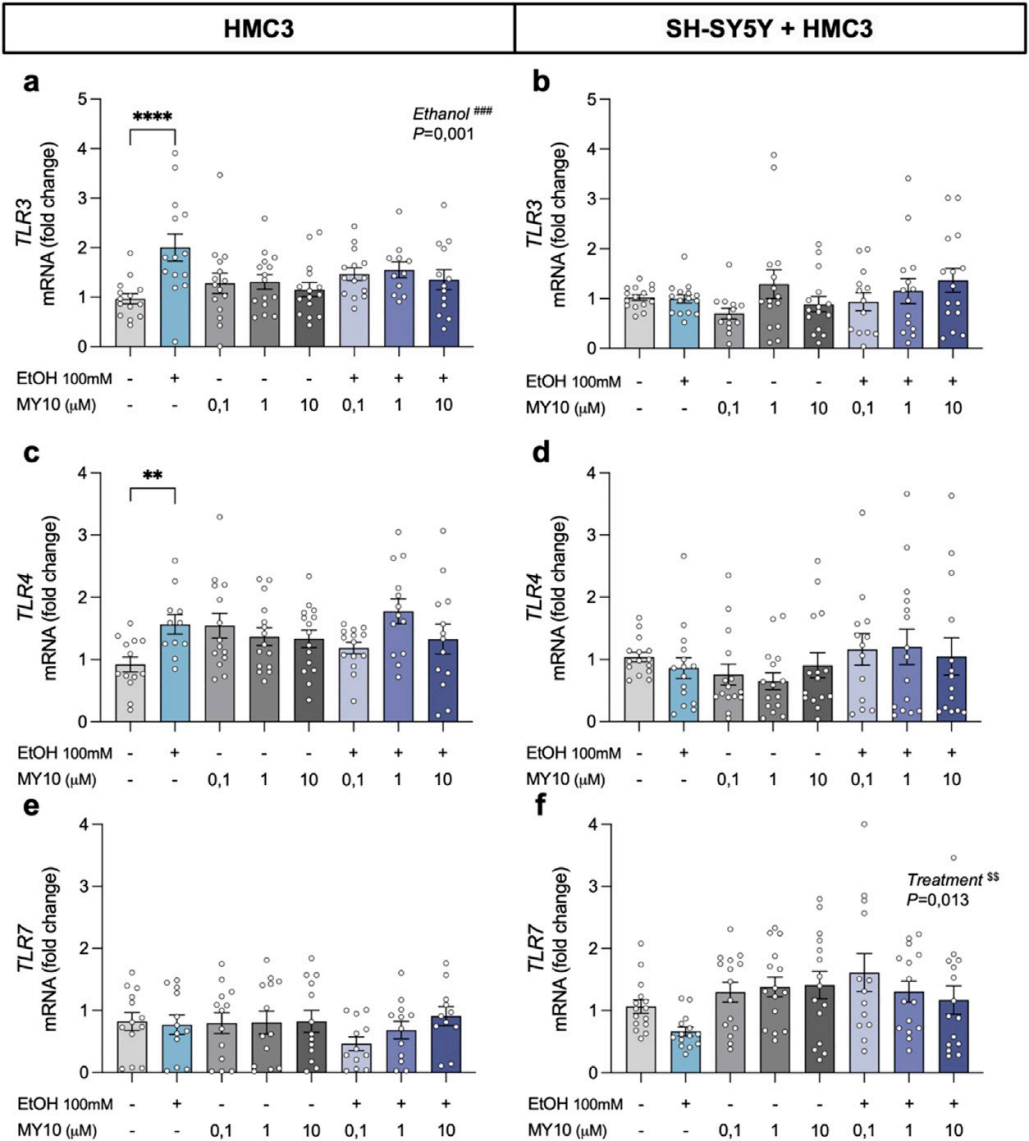
Effect of 24-h ethanol exposure (100 mM) and MY10 treatment on the expression of Toll-like receptor (TLRs) genes in microglia and co-cultures of neurons and microglia. *TLR3* mRNA expression in HMC3 cells **(a)** and microglial-neuron co-culture **(b)**. *TLR4* mRNA expression in HMC3 cells **(c)** and microglial-neuron co-culture **(d)**. *TLR7* mRNA expression in HMC3 cells **(e)** and microglial-neuron co-culture **(f)**. Data are presented as mean ± SEM. **P < 0.01, ****P < 0.0001. ^###^ *P* ≤ 0.001 for a significant effect of EtOH exposure. ^$$^ *P* ≤ 0.01 for significant effect of MY10 treatment. EtOH: ethanol.

To further explore the pathways involved in ethanol-induced neuroinflammation, we evaluated the transcriptional profiles of Toll-like receptors (TLRs) (Figure 4). The two-way ANOVA revealed a significant effect of ethanol exposure on HMC3 cells (*F* (1,103) = 10.8; *P* = 0.0014) and a significant interaction between ethanol and treatment with MY10 (*F* (3,103) = 2.705; *P* < 0.05) on *TLR3* mRNA expression. Ethanol exposure upregulated *TLR3* mRNA expression levels in microglial cells, an effect that was attenuated by treatment with MY10 (Figure 4a). In contrast, we did not find significant effects of ethanol exposure or treatment with MY10 in the expression of *TLR3* in the co-culture of microglia and neurons (Figure 4b). Ethanol exposure increased *TLR4* mRNA expression levels in HMC3 cells with a significant interaction between ethanol and treatment with MY10 (*F* (3,100) = 3.455; *P* = 0.0193) (Figure 4c). As in the case of *TLR3*, this effect was not observed in the co-cultures (Figure 4d). We did not find significant effects of ethanol exposure or treatment with MY10 in the expression of *TLR7* in HMC3 cells (Figure 4e). In the co-cultures, the two-way ANOVA performed revealed a significant effect of the treatment with MY10 (*F* (3,109) = 3.757; *P* = 0.013), which caused a modest but consistent increase on *TLR7* mRNA expression levels in all groups treated with MY10 independently of the concentration (Figure 4f).

## 4 Discussion

In this study, we used a human co-culture model that combines HMC3 microglial cells with SH-SY5Y cells differentiated into neurons. This model was developed to create a physiologically relevant platform (Goshi et al., 2020) for studying ethanol-induced neuroinflammatory responses and the possible modulatory role of RPTPβ/ζ.

We observed that ethanol selectively modulates the expression of *PTN* and *MK* depending on the cellular context. Interestingly, *PTN* expression was upregulated in HMC3 monocultures following ethanol exposure, whereas *MK* was downregulated in co-cultures. These differences suggest that microglia respond to ethanol by increasing the expression of a potent neurotrophic factor, *PTN*, suggesting a stress-adaptive response in these critical immune cells. In contrast, the decreased expression of *MK* induced by ethanol exposure in co-cultures suggests a complex regulation of this neurotrophic factor expression involving neuronal-microglial interactions. These results are important since the cerebral expression of both *PTN* and MK is regulated by ethanol in both humans and animal models (Herradon et al., 2019). Our data indicate for the first time that ethanol exposure differentially regulated *PTN* and *MK* expression depending on the cell type and neuron-microglia communication.

In addition, we observed a robust microglial response induced by ethanol exposure, characterized by increased expression of proinflammatory genes such as *CCL2* and *TLRs*, consistent with previous reports (Crews et al., 2011; Qin and Crews, 2012; Coleman et al., 2017; Walter and Crews, 2017; Lawrimore and Crews, 2017; Lawrimore et al., 2019). In contrast, neurons in monoculture exhibited a downregulation of *CCL2* expression alongside a pronounced upregulation of *iNOS*, suggesting a limited but specific response to ethanol exposure. These neurons appear to engage selective stress-related signaling pathways in response to the neurotoxic stimulus (Lawrimore and Crews, 2017).

When both cell populations were integrated into a co-culture system, we observed a substantial attenuation of ethanol-induced inflammatory responses compared to microglia monocultures (Abellanas et al., 2019; Haenseler et al., 2017), reflected in the expression of key proinflammatory markers, such as *CCL2, IL-1β,* as well as *TLR3* and *TLR4*. These findings suggest that neurons exert a dampening effect on microglial reactivity, potentially through direct cell-to-cell signaling. This interaction may have important implications for regulating neuroimmune responses and preserving neuronal viability (Alfonso-Loeches et al., 2010; Crews et al., 2017; Correa et al., 2013; Kraft and Harry, 2011). Our results align with previous co-culture studies that have demonstrated the cross-regulation of glial reactivity via neuronal signals (Crews et al., 2024; Zou and Crews, 2014; Lawrimore et al., 2019; Boyadjieva and Sarkar, 2010; Arzua et al., 2020).

The pharmacological inhibition of RPTPβ/ζ with MY10 revealed a selective and cell-dependent modulation of neuroinflammatory signaling. In both SH-SY5Y and HMC3 monocultures, MY10 treatment alone did not alter the expression of most proinflammatory genes, suggesting that basal activation of this pathway may be limited in cellular resting states. However, in cells exposed to ethanol, MY10 partially influenced the inflammatory responses by regulating the expression of specific genes, such as *IL-1β* and *TNFα*, in both microglial cells and co-cultures. Moreover, MY10 significantly altered the *TLR7* expression in co-cultures, indicating that its effects are not limited to cytokine modulation but may extend to upstream components of innate immune signaling (Coleman and Crews, 2018). These results underscore the importance of studying pharmacological interventions in models that more accurately reflect *in vivo* cellular communication (Coleman et al., 2017; Coleman and Crews, 2018). Furthermore, these findings support previous studies using animal models regarding the neuroprotective and anti-inflammatory properties of MY10, validating the PTN/MK–RPTPβ/ζ signaling axis as a promising therapeutic target in ethanol-induced brain injury (Calleja-Conde et al., 2020; Fernández-Calle et al., 2018; Fernández-Calle et al., 2019; Fernández-Calle et al., 2020; Galán-Llario et al., 2023a; Galán-Llario et al., 2023b; Herradon et al., 2019).

A point to consider in the present work is the lack of protein-level validation of the transcriptional changes observed. Although gene expression analysis provides a valuable overview of regulatory trends, post-transcriptional mechanisms may alter final protein levels. Future studies quantifying key neuroimmune mediators, such as CX3CL1 (Fractalkine), should be useful to validate and complement the transcriptomic data and further elucidate the bidirectional signaling between neurons and microglia in response to alcohol and MY10. In addition, it is important to note that a limitation of this study is the inability to determine the specific cellular origin of gene expression changes in the co-culture system. However, as in many *in vivo* murine models that analyze entire brain regions without isolating specific cell types, this approach reflects the complexity of the brain environment and captures coordinated neuroimmune responses. It provides biologically significant, translationally relevant insights and facilitates the future application of cell-specific techniques such as immunocytochemistry, flow cytometry or single-cell RNA sequencing (scRNA-seq), which allow higher-resolution analysis of heterogeneous systems. Recent studies using scRNA-seq in alcohol dependence models (Salem et al., 2024; Warden et al., 2024) have demonstrated the utility of this approach in resolving cell-type and region-specific transcriptomic changes. In fact, it has allowed us to interpret more accurately the data collected here in human cells in the context of the relevant previous studies that defined the effects of MY10 and ethanol in different rodent models (Calleja-Conde et al., 2020; Fernández-Calle et al., 2018; Galán-Llario et al., 2023a; Galán-Llario et al., 2023b). In this sense, previous studies using primary rodent cultures of both cortical and hippocampal neurons and microglia have demonstrated strong inflammatory responses to ethanol and other neurotoxic agents (Crews et al., 2011; Coleman et al., 2017). However, species-specific differences in aspects such as alcohol metabolism, gene expression, and immune response limit the translational relevance of these models (Pascual et al., 2018; Walter and Crews, 2017; Seok et al., 2013). Additionally, reliance exclusively on murine models may fail to fully identify human-specific regulation of genes in response to alcohol, as revealed by comparative transcriptomic analyses of postmortem human and mouse brain tissue (Pascual et al., 2018; van der Worp et al., 2010). Our study suggests that human co-culture systems derived from immortalized cell lines offer a level of standardization that is difficult to achieve with primary cultures, often limited by heterogeneity and availability (Abud et al., 2017; Dello Russo et al., 2018; Pascual et al., 2018; Walter and Crews, 2017; Szabo and Gulya, 2013).

In conclusion, our study provides novel insights into the modulatory actions of microglial-neuronal interactions in ethanol-induced neuroimmune responses. These findings strongly support the utility of the human neuronal-microglial co-culture model as a physiologically relevant *in vitro* platform for studying ethanol-induced immune responses. Furthermore, the pharmacological assessment of the RPTPβ/ζ inhibitor MY10 in this context highlights the potential of this signaling pathway as an innovative therapeutic target in the deleterious effects of ethanol in the brain. While the model has inherent limitations due to the use of 2D systems and immortalized cell lines, it offers a valuable approach for advancing translational research on alcohol pathophysiology and the development of novel neuroprotective strategies.

## Data Availability

The datasets are available from the corresponding author upon request.

## References

[B1] Abellanas M. A. Zamarbide M. Basurco L. Luquin E. Garcia-Granero M. Clavero P. (2019). Midbrain microglia mediate a specific immunosuppressive response under inflammatory conditions. J. Neuroinflammation 16, 233. 10.1186/s12974-019-1628-8 31757220 PMC6874825

[B2] Abud E. M. Ramirez R. N. Martinez E. S. Healy L. M. Nguyen C. H. H. Newman S. A. (2017). iPSC-Derived human microglia-like cells to study neurological diseases. Neuron 94 (2), 278–293. 10.1016/j.neuron.2017.03.042 28426964 PMC5482419

[B3] Alfonso-Loeches S. Pascual-Lucas M. Blanco A. M. Sanchez-Vera I. Guerri C. (2010). Pivotal role of TLR4 receptors in alcohol-induced neuroinflammation and brain damage. J. Neurosci. official J. Soc. Neurosci. 30 (24), 8285–8295. 10.1523/JNEUROSCI.0976-10.2010 20554880 PMC6634595

[B4] Arzua T. Yan Y. Jiang C. Logan S. Allison R. L. Wells C. (2020). Modeling alcohol-induced neurotoxicity using human induced pluripotent stem cell-derived three-dimensional cerebral organoids. Transl. psychiatry 10 (1), 347. 10.1038/s41398-020-01029-4 33051447 PMC7553959

[B5] Boyadjieva N. I. Sarkar D. K. (2010). Role of microglia in ethanol's apoptotic action on hypothalamic neuronal cells in primary cultures. Alcohol. Clin. Exp. Res. 34 (11), 1835–1842. 10.1111/j.1530-0277.2010.01271.x 20662807 PMC2965273

[B6] Calleja-Conde J. Fernández-Calle R. Zapico J. M. Ramos A. de Pascual-Teresa B. Bühler K. M. (2020). Inhibition of receptor protein tyrosine phosphatase β/ζ reduces alcohol intake in rats. Alcohol. Clin. Exp. Res. 44 (5), 1037–1045. 10.1111/acer.14321 32154588

[B7] Coleman L. G. Jr Crews F. T. (2018). Innate immune signaling and alcohol use disorders. Handb. Exp. Pharmacol. 248, 369–396. 10.1007/164_2018_92 29500721 PMC6120815

[B8] Coleman L. G. Jr Zou J. Crews F. T. (2017). Microglial-derived miRNA let-7 and HMGB1 contribute to ethanol-induced neurotoxicity *via* TLR7. J. neuroinflammation 14 (1), 22. 10.1186/s12974-017-0799-4 28118842 PMC5264311

[B9] Coleman L. G. Jr Zou J. Qin L. Crews F. T. (2018). HMGB1/IL-1β complexes regulate neuroimmune responses in alcoholism. Brain, Behav. Immun. 72, 61–77. 10.1016/j.bbi.2017.10.027 29102800 PMC5932292

[B10] Correa F. G. Hernangómez M. Guaza C. (2013). Understanding microglia-neuron cross talk: relevance of the microglia-neuron cocultures. Methods Mol. Biol. Clift. N.J. 1041, 215–229. 10.1007/978-1-62703-520-0_20 23813382

[B11] Crews F. T. (2012). Immune function genes, genetics, and the neurobiology of addiction. Alcohol Res. Curr. Rev. 34 (3), 355–361. 10.17615/3882-nr29 23134052 PMC3860409

[B12] Crews F. T. Coleman L. G. Jr Macht V. A. Vetreno R. P. (2024). Alcohol, HMGB1, and innate immune signaling in the brain. Alcohol Res. Curr. Rev. 44 (1), 04. 10.35946/arcr.v44.1.04 39135668 PMC11318841

[B13] Crews F. T. Walter T. J. Coleman L. G. Jr Vetreno R. P. (2017). Toll-like receptor signaling and stages of addiction. Psychopharmacology 234 (9-10), 1483–1498. 10.1007/s00213-017-4560-6 28210782 PMC5420377

[B14] Crews F. T. Zou J. Qin L. (2011). Induction of innate immune genes in brain create the neurobiology of addiction. Brain, Behav. Immun. 25 (Suppl. 1), S4-S12–S12. 10.1016/j.bbi.2011.03.003 21402143 PMC3552373

[B15] Del Campo M. Fernández-Calle R. Vicente-Rodríguez M. Martín Martínez S. Gramage E. Zapico J. M. (2021). Role of receptor protein tyrosine phosphatase β/ζ in neuron-microglia communication in a cellular model of parkinson's disease. Int. J. Mol. Sci. 22 (13), 6646. 10.3390/ijms22136646 34206170 PMC8269034

[B16] Dello Russo C. Cappoli N. Coletta I. Mezzogori D. Paciello F. Pozzoli G. (2018). The human microglial HMC3 cell line: where do we stand? A systematic literature review. J. neuroinflammation 15 (1), 259. 10.1186/s12974-018-1288-0 30200996 PMC6131758

[B17] Fernández-Calle R. Galán-Llario M. Gramage E. Zapatería B. Vicente-Rodríguez M. Zapico J. M. (2020). Role of RPTPβ/ζ in neuroinflammation and microglia-neuron communication. Sci. Rep. 10 (1), 20259. 10.1038/s41598-020-76415-5 33219280 PMC7679445

[B18] Fernández-Calle R. Gramage E. Zapico J. M. de Pascual-Teresa B. Ramos A. Herradón G. (2019). Inhibition of RPTPβ/ζ blocks ethanol-induced conditioned place preference in pleiotrophin knockout mice. Behav. brain Res. 369, 111933. 10.1016/j.bbr.2019.111933 31054277

[B19] Fernández-Calle R. Vicente-Rodríguez M. Gramage E. Pita J. Pérez-García C. Ferrer-Alcón M. (2017). Pleiotrophin regulates microglia-mediated neuroinflammation. J. neuroinflammation 14 (1), 46. 10.1186/s12974-017-0823-8 28259175 PMC5336633

[B20] Fernández-Calle R. Vicente-Rodríguez M. Pastor M. Gramage E. Di Geronimo B. Zapico J. M. (2018). Pharmacological inhibition of receptor protein tyrosine phosphatase β/ζ (PTPRZ1) modulates behavioral responses to ethanol. Neuropharmacology 137, 86–95. 10.1016/j.neuropharm.2018.04.027 29753117 PMC6050104

[B21] Galán-Llario M. Gramage E. García-Guerra A. Torregrosa A. B. Gasparyan A. Navarro D. (2024). Adolescent intermittent ethanol exposure decreases perineuronal nets in the hippocampus in a sex dependent manner: modulation through pharmacological inhibition of RPTPβ/ζ. Neuropharmacology 247, 109850. 10.1016/j.neuropharm.2024.109850 38295947

[B22] Galán-Llario M. Rodríguez-Zapata M. Fontán-Baselga T. Gramage E. Vicente-Rodríguez M. Zapico J. M. (2023b). Inhibition of RPTPβ/ζ reduces chronic ethanol intake in adolescent mice and modulates ethanol effects on hippocampal neurogenesis and glial responses in a sex-dependent manner. Neuropharmacology 227, 109438. 10.1016/j.neuropharm.2023.109438 36706907 PMC10327582

[B23] Galán-Llario M. Rodríguez-Zapata M. Gramage E. Vicente-Rodríguez M. Fontán-Baselga T. Ovejero-Benito M. C. (2023a). Receptor protein tyrosine phosphatase β/ζ regulates loss of neurogenesis in the mouse hippocampus following adolescent acute ethanol exposure. Neurotoxicology 94, 98–107. 10.1016/j.neuro.2022.11.008 36402194

[B24] Goshi N. Morgan R. K. Lein P. J. Seker E. (2020). A primary neural cell culture model to study neuron, astrocyte, and microglia interactions in neuroinflammation. J. neuroinflammation 17 (1), 155. 10.1186/s12974-020-01819-z 32393376 PMC7216677

[B25] Gresa-Arribas N. Viéitez C. Dentesano G. Serratosa J. Saura J. Solà C. (2012). Modelling neuroinflammation *in vitro:* a tool to test the potential neuroprotective effect of anti-inflammatory agents. PloS one 7 (9), e45227. 10.1371/journal.pone.0045227 23028862 PMC3447933

[B26] Haenseler W. Sansom S. N. Buchrieser J. Newey S. E. Moore C. S. Nicholls F. J. (2017). A highly efficient human pluripotent stem cell microglia model displays a neuronal-co-culture-specific expression profile and inflammatory response. Stem cell Rep. 8 (6), 1727–1742. 10.1016/j.stemcr.2017.05.017 28591653 PMC5470330

[B27] Hartung T. (2009). Toxicology for the twenty-first century. Nature 460 (7252), 208–212. 10.1038/460208a 19587762

[B28] Herradón G. Pérez-García C. (2014). Targeting midkine and pleiotrophin signalling pathways in addiction and neurodegenerative disorders: recent progress and perspectives. Br. J. Pharmacol. 171 (4), 837–848. 10.1111/bph.12312 23889475 PMC3925022

[B29] Herradon G. Ramos-Alvarez M. P. Gramage E. (2019). Connecting metainflammation and neuroinflammation through the PTN-MK-RPTPβ/ζ axis: relevance in therapeutic development. Front. Pharmacol. 10, 377. 10.3389/fphar.2019.00377 31031625 PMC6474308

[B30] Kraft A. D. Harry G. J. (2011). Features of microglia and neuroinflammation relevant to environmental exposure and neurotoxicity. Int. J. Environ. Res. public health 8 (7), 2980–3018. 10.3390/ijerph8072980 21845170 PMC3155341

[B31] Lawrimore C. J. Coleman L. G. Zou J. Crews F. T. (2019). Ethanol induction of innate immune signals across BV2 microglia and SH-SY5Y neuroblastoma involves induction of IL-4 and IL-13. Brain Sci. 9 (9), 228. 10.3390/brainsci9090228 31510019 PMC6770440

[B32] Lawrimore C. J. Crews F. T. (2017). Ethanol, TLR3, and TLR4 agonists have unique innate immune responses in neuron-like SH-SY5Y and microglia-like BV2. Alcohol. Clin. Exp. Res. 41 (5), 939–954. 10.1111/acer.13368 28273337 PMC5407472

[B33] Pascual M. López-Hidalgo R. Montagud-Romero S. Ureña-Peralta J. R. Rodríguez-Arias M. Guerri C. (2021). Role of mTOR-regulated autophagy in spine pruning defects and memory impairments induced by binge-like ethanol treatment in adolescent mice. Brain pathol. 31 (1), 174–188. 10.1111/bpa.12896 32876364 PMC8018167

[B34] Pascual M. Montesinos J. Guerri C. (2018). Role of the innate immune system in the neuropathological consequences induced by adolescent binge drinking. J. Neurosci. Res. 96 (5), 765–780. 10.1002/jnr.24203 29214654

[B35] Pastor M. Fernández-Calle R. Di Geronimo B. Vicente-Rodríguez M. Zapico J. M. Gramage E. (2018). Development of inhibitors of receptor protein tyrosine phosphatase β/ζ (PTPRZ1) as candidates for CNS disorders. Eur. J. Med. Chem. 144, 318–329. 10.1016/j.ejmech.2017.11.080 29275231 PMC5817915

[B36] Qin L. Crews F. T. (2012). NADPH oxidase and reactive oxygen species contribute to alcohol-induced microglial activation and neurodegeneration. J. neuroinflammation 9, 5. 10.1186/1742-2094-9-5 22240163 PMC3271961

[B37] Rodríguez-Zapata M. Galán-Llario M. Cañeque-Rufo H. Sevillano J. Sánchez-Alonso M. G. Zapico J. M. (2023). Implication of the PTN/RPTPβ/ζ signaling pathway in acute ethanol neuroinflammation in both sexes: a comparative study with LPS. Biomedicines 11 (5), 1318. 10.3390/biomedicines11051318 37238989 PMC10215719

[B38] Roqué P. J. Costa L. G. (2017). Co-Culture of neurons and microglia. Curr. Protoc. Toxicol. 74, 11.24.1–11.24.17. 10.1002/cptx.32 29117434 PMC5774987

[B39] Salem N. A. Manzano L. Keist M. W. Ponomareva O. Roberts A. J. Roberto M. (2024). Cell-type brain-region specific changes in prefrontal cortex of a mouse model of alcohol dependence. Neurobiol. Dis. 190, 106361. 10.1016/j.nbd.2023.106361 37992784 PMC10874299

[B40] Seok J. Warren H. S. Cuenca A. G. Mindrinos M. N. Baker H. V. Xu W. (2013). Genomic responses in mouse models poorly mimic human inflammatory diseases. Proc. Natl. Acad. Sci. U. S. A. 110 (9), 3507–3512. 10.1073/pnas.1222878110 23401516 PMC3587220

[B41] Szabo M. Gulya K. (2013). Development of the microglial phenotype in culture. Neuroscience 241, 280–295. 10.1016/j.neuroscience.2013.03.033 23535251

[B42] van der Worp H. B. Howells D. W. Sena E. S. Porritt M. J. Rewell S. O'Collins V. (2010). Can animal models of disease reliably inform human studies? PLoS Med. 7 (3), e1000245. 10.1371/journal.pmed.1000245 20361020 PMC2846855

[B43] Walter T. J. Crews F. T. (2017). Microglial depletion alters the brain neuroimmune response to acute binge ethanol withdrawal. J. Neuroinflammation 14, 86. 10.1186/s12974-017-0856-z 28427424 PMC5439231

[B44] Warden A. S. Salem N. A. Brenner E. Sutherland G. T. Stevens J. Kapoor M. (2024). Integrative genomics approach identifies glial transcriptomic dysregulation and risk in the cortex of individuals with alcohol use disorder. bioRxiv Prepr. Serv. Biol. 2024, 2024.08.16.607185. 10.1101/2024.08.16.607185 40024496 PMC12667600

[B45] Zou J. Y. Crews F. T. (2014). Release of neuronal HMGB1 by ethanol through decreased HDAC activity activates brain neuroimmune signaling. PloS one 9 (2), e87915. 10.1371/journal.pone.0087915 24551070 PMC3925099

