# Supplementary material for: Ethanol-induced changes in neurotrophic and immune genes are regulated by receptor-type protein tyrosine phosphatase β/ζ (RPTPβ/ζ) and microglial-neuronal interactions
Source: Front Genet. 2025 Aug 22;16:1634202. doi: 10.3389/fgene.2025.1634202 (PMC12412308; doi:10.3389/fgene.2025.1634202)
Supplement: Supplementary file 1 [file DataSheet1.docx]

Supplementary Material

## Supplementary Figure 1


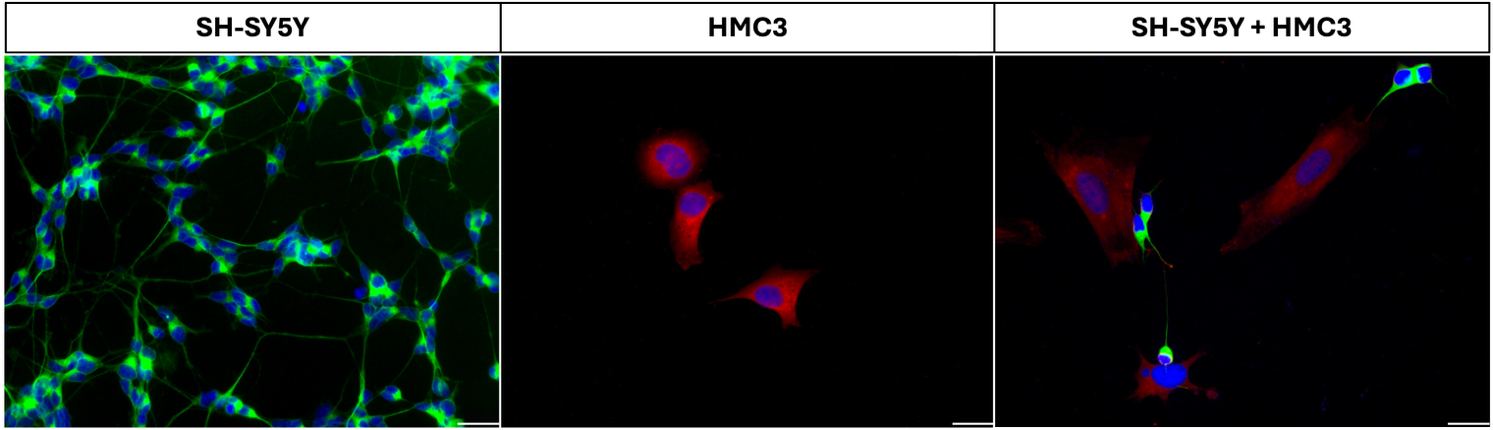


**Supplementary Figure 1. Fluorescence Immunocytochemistry of cultures:** SH-SY5Y cells differentiated to a dopaminergic phenotype, HMC3 and co-culture of both cells, labelled with DAPI (blue), MAP-2 (green), and IBA1 (red). Scale bar 25 µm.
